# Biogeography of the Respiratory Tract Microbiome in Patients With Malignant Tracheal Tumors

**DOI:** 10.3389/fonc.2021.758917

**Published:** 2021-11-19

**Authors:** Kai-Xiong Liu, Hai-Xia Liu, Jing Zhang, Nan Zhang, Yun-Zhi Zhou, Mei-Mei Tao, Hong-Wu Wang, Jie-Ming Qu

**Affiliations:** ^1^ Department of Respiratory and Critical Care Medicine, Ruijin Hospital, School of Medicine, Shanghai Jiao Tong University, Shanghai, China; ^2^ Institute of Respiratory Diseases, School of Medicine, Shanghai Jiao Tong University, Shanghai, China; ^3^ Shanghai Key Laboratory of Emergency Prevention Diagnosis and Treatment of Respiratory Infectious Disease, Shanghai, China; ^4^ Department of Respiratory and Critical Care Medicine, The First Affiliated Hospital, Fujian Medical University, Fuzhou, China; ^5^ Department of Infectious Disease, Zhongshan Hospital, Fudan University, Shanghai, China; ^6^ Department of Respiratory and Critical Care Medicine, Zhongshan Hospital, Fudan University, Shanghai, China; ^7^ Department of Oncology, Emergency General Hospital, Beijing, China; ^8^ Department of Respiratory Medicine, Emergency General Hospital, Beijing, China; ^9^ Department of Respiratory and Critical Care Medicine, Dongzhimen Hospital Affiliated to Beijing University of Chinese Medicine, Beijing, China

**Keywords:** malignant tracheal tumor, squamous cell carcinoma, salivary gland type carcinoma, 16S rRNA sequencing, microbiome

## Abstract

**Background:**

This study aimed to characterize the bacterial microbiota in the oral cavity (OC), throat, trachea, and distal alveoli of patients with primary malignant tracheal tumors (PMTT), including squamous cell carcinoma (SCC) and salivary gland carcinoma patients (SGC), for comparison with a matched non-malignant tracheal tumor (NMTT) group.

**Methods:**

Patients with pathological diagnosis of PMTT and NMTT were included in this study. Saliva, throat swab (TS), trachea protected specimen brush (PSB), and bronchoalveolar lavage fluid (BALF) samples were collected for 16S rRNA gene sequencing. The composition, diversity, and distribution of the microbiota were compared among biogeographic sampling sites and patient groups. The relationship between the genera-level taxon abundance and tracheal tumor types was also investigated to screen for candidate biomarkers.

**Findings:**

The most represented phyla in the four sites were Bacteroidetes, Firmicutes, Proteobacteria, and Fusobacteria. In SCC patients, the relative abundance of *Bacteroidetes* and *Firmicutes* gradually decreased with increasing depth into the respiratory tract, while the relative abundance of *Proteobacteria* gradually increased. Bacterial communities at the four biogeographic sites formed two distinct clusters, with OC and TS samples comprising one cluster and PSB and BALF samples comprising the other group. Principal coordinate analysis showed that trachea microbiota in SCC patients were distinct from that of SGC or NMTT patients. In the trachea, AUCs generated by *Prevotella* and *Alloprevotella* showed that the abundance of these genera could distinguish SCC patients from both NMTT and SGC patients.

**Interpretation:**

The structure of respiratory tract microbiota in PMTT patients is related to tumor type. Certain bacteria could potentially serve as markers of SCC, although verification with large-sample studies is necessary.

## Introduction

Primary malignant tracheal tumor (PMTT) is a rare disease, accounting for 0.5% of all malignancies and 0.01-0.4% of lung cancer cases, with approximately 1 new case per 1,000,000 reported yearly (1, 2). Histologically, PMTT mainly includes squamous cell carcinoma (SCC), and salivary gland type carcinoma (SGC) (3). Although PMTT represents a small proportion of total lung cancer cases, a large number of patients suffer from PMTT. PMTT most commonly arise from the respiratory epithelium, mesenchymal structures, or salivary glands of the trachea (3). The possible factors affecting the occurrence and development of tracheal cancer include underlying genetics, smoking, air pollution and other environmental factors. However, the exact mechanism of PMTT development has not been determined.

The lung has been historically considered a sterile environment. However, recent advances in high-throughput sequencing have shed new light on this misconception with evidence that there are abundant and diverse bacterial communities in the lower respiratory tract (LRT) dominated by Bacteroidetes, Firmicutes, and Proteobacteria phyla (4, 5). Several studies have shown that microbiota of the human lung contribute significantly to respiratory health and disease (5–7). Specifically, alterations in the composition and diversity of respiratory tract microbiota are common in lung diseases such as asthma (8), lung fibrosis (9), lung cancers (7, 10–15), and chronic obstructive pulmonary disease (16). However, respiratory microbiota is relatively unstudied compared to gut microbiota, and the tracheal microbiome under disease conditions is less studied than that in healthy subjects or subjects with laryngotracheal stenosis (17, 18). The trachea is regularly exposed to air pollution, particulate matter, tobacco smoke, and microbes, all of which can either cause or contribute to microbial dysbiosis and inflammation, consequently impacting tumorigenic processes. Therefore, further exploration of the relationship between microbiota and PMTT is needed to better understand their underlying interactions.

To date, there has been no report of which we are aware describing the composition and diversity of respiratory tract microbiota in PMTT patients. In this study, we used 16s rRNA gene sequencing to characterize the respiratory tract microbiota in patients with PMTT and compared their biogeographic distribution in the upper respiratory tract (URT), oral cavity (OC) and LRT. We hypothesized that tracheal tumors would be associated with respiratory tract dysbiosis and microbial populations will exhibit changes in their relative abundance along the respiratory tract in patients with PMTT compared with that in non-malignant tracheal tumor (NMTT) patients. We then identified significant differences in airway microbiota composition and characteristics among patients with NMTT, SGC, and SCC, as well as between different sites in the respiratory tract. In addition, we looked for correlations between specific taxa and disease states at each site to screen for candidate bacterial biomarkers associated with PMTT. Our findings provide a basis for exploration of the role of microorganisms in PMTT, as well as potential biomarkers for further development in clinical diagnostics.

## Methods

### Ethical Statement

This study was conducted in accordance with standards in the Declaration of Helsinki (as revised in 2013). This study was approved by the Ethics Committee of Ruijin Hospital Affiliated with Shanghai Jiaotong University (2017-NO-81) and Emergency General Hospital (K202110). Written informed consent was obtained from all participants.

### Study Design

This prospective observational study was conducted in Ruijin Hospital Affiliated with Shanghai Jiaotong University and Emergency General Hospital from March 2018 to March 2020. Patients with histologically confirmed PMTT and NMTT were recruited for this study. Included subjects were limited to patients aged 18-79 years at diagnosis and with a survival time of ≥ 3 months. Subjects were excluded if they had any of the following conditions: prior history of cancer, tuberculosis, recent bacterial/viral respiratory infection (within 3 months), cirrhosis, diabetes, pregnancy, endotracheal intubation, current smoking or smoking cessation for less than three months, use of probiotics and antibiotics (within the past three months), or contraindications to bronchoscopy. Data for clinical characteristics were obtained by questionnaires and medical charts.

### Sample Collection

Samples from OC saliva, throat swab (TS), trachea protected specimens brush (PSB), and bronchoalveolar lavage fluid (BALF) were collected for 16S rRNA gene analysis. Saliva and TS samples were collected before bronchoscopy diagnosis. Bronchoscopy was performed by experienced physicians. The bronchoscope was inserted into the mouth and secured in a position to obtain tracheal PSB and BALF samples. PSB samples were collected prior to Bronchoalveolar lavage (BAL). BALF samples were collected according to American Thoracic Society guidelines and Chinese Thoracic Society standard procedures (19). All samples were transported to the laboratory using dry ice buckets and stored at -80°C.

### DNA Extraction and High-Throughput Sequencing

All samples were subjected to the same procedures for DNA extraction and PCR amplification, which were performed by the same laboratory technicians. Microbial DNA was extracted from samples as previously described (20). The extracted DNA from each sample was used as the template for PCR amplification of the V3~V4 region of the bacterial 16S rRNA gene, using primers F1 (5’-CCTACGGGNGGCWGCAG-3’) and R2 (5’-GACTACHVGvGGTATCTAATCC-3’) which corresponds to positions 341 to 805 in the *Escherichia coli* 16S rRNA gene. PCR reactions were run in an EasyCycler 96 PCR system (Analytik Jena Corp., AG) using the following program: 3 min of denaturation at 95°C; 21 cycles of 0.5 min at 94°C (denaturation), 0.5 min 58°C (annealing), and 0.5 min at 72°C (elongation); 5 min final extension at 72°C. The products from all samples were indexed and mixed at equal ratios for sequencing by Shanghai Mobio Biomedical Technology Co. Ltd. using the Miseq platform (Illumina Inc., USA) according to the manufacturer’s instructions.

We used a previously described set of minimal experimental criteria to minimize the impact of contaminant DNA and likelihood of cross-contamination during research (21). To identify potential sources of contamination in sequencing, we collected multiple procedural controls, including saline used in bronchoscopy, unused protected specimens brush, and sterile water used in library.

### Reads Processing

Clean reads were extracted from raw data using USEARCH (version 11.0.667) with the following criteria: (i) Sequences of each sample were extracted using each index with zero mismatches. (ii) Sequences with overlap of less than 50 bp were discarded. (iii) Sequences with an error rate for overlap greater than 0.1 were discarded. (iv) Sequences less than 400 bp after merging were discarded. Quality-filtered sequences were clustered into unique sequences and sorted in order of decreasing abundance to identify representative sequences using UPARSE according to the UPARSE OTU analysis pipeline; singletons were omitted in this step.

### Bioinformatics Analysis of 16S rRNA

Operational Taxonomic Units (OTUs) were classified based on 97% similarity after chimeric sequences removed using UPARSE (version 7.1 http://drive5.com/uparse/). The phylogenetic affiliation of each 16S rRNA gene sequence was analyzed by RDP Classifier (http://rdp.cme.msu.edu/) against the Silva database (SSU123) using a confidence threshold of 70% (22). Alpha diversity was assessed using the ACE estimator, Chao 1 estimator, Shannon-Wiener diversity index, and Simpson diversity index. Both Bray-Curtis, weighted and unweighted UniFrac dissimilarity were calculated in QIIME (23). Principal coordinate analysis (PCoA) plots and PERMANOVA were used to test for statistical significance between the groups using 10,000 permutations using the adonis function of vegan 2.5-7 (24). Differences in bacterial population structure between different sites were inspected using the anosim function (vegan package) with 1,000 permutations. The linear discriminant analysis (LDA) effect size (LEfSe) was used to detect taxa with differential abundance among groups (lefse 1.1, https://github.com/SegataLab/lefse). Raw Illumina read data for all samples were deposited in the GenBank Sequence Read Archive under accession number (PRJNA766788). Microbiome statistics and figure generation were performed in R 4.0 using the ggplot2 package (25).

### Biomarker Prediction and Screening

We constructed a random forest model for distinguishing and classification of predominant genera as candidate biomarkers based on Gini importance values for OTUS between groups using the R package function “randomForest” (ntree=1000) (26). The discriminatory ability of each candidate biomarker was evaluated by plotting receiver-operating characteristic (ROC) curves and calculating the area under the ROC curve (AUC) using the pROC package in the R software package. Due to the small sample size, we only performed classification and did not perform validation with a separate cohort.

### Statistical Analysis

Continuous variables were reported as means ± standard deviations, and statistical comparisons were made using the independent t-test. Non-normally distributed variables were expressed as interquartile range (IQR), and comparisons were conducted using the Mann-Whitney U test. Differences with a *p* value <0.05 (two-sided) were considered statistically significant. Statistical analyses were performed using SPSS V.20.0 (SPSS, Chicago, Illinois, USA).

## Results

### Baseline Clinical Characteristics of the Participants

A total of 55 patients with PMTT (n=34) or NMTT (n=21) were recruited. The demographic and clinical data of all the participants are shown in **Table 1**. PMTT participants were then divided into SGC (n = 15) or SCC (n = 19) groups depending on carcinoma type. The NMTT group consisted of patients with tracheal adenoma (n=6), hamartoma (n=6), lipoma (n=1), leiomyoma (n=3), papilloma (n=3), or idiopathic glottic stenosis (n=2). There were no significant differences in age, body mass index (BMI), or smoking status among the groups. Of these patients, 54 consented to bronchoscopy. It should also be noted that DNA extraction was unsuccessful in 12 samples.

**Table 1:** Demographic and clinical characteristics of the patients.

	SCC (n = 19)	SGC (n = 15)	NMTT (n = 21)	*P* value
Age ± SD	60.40 ± 2.37	51.46 ± 4.25	54.00 ± 3.22	0.194
Gender: male (n)	9	5	10	
Smoking Status				
Past Smoker (n)	10	6	9	0.729
Never Smoker (n)	9	9	12	0.729
Metastasis (n)	4	2	–	0.558
Location in trachea				
Cervical (n)	2	5	5	0.869
Middle (n)	7	6	7	
Distal and carinal (n)	10	4	9	
Samples passing quality control/samples sequenced				
Saliva (n)	16	15	21	–
Throat (n)	19	13	17	–
Trachea (n)	16	15	21	–
BALF (n)	19	15	21	–

One-way analysis of variance was used to evaluate the difference among the three groups. Continuous variables were compared using Wilcoxon rank sum test between both groups. Fisher’s exact test compared categorical variables.

### Taxonomic Profiling of Tracheal Tumor Airway Microbiota

**Figure 1:**
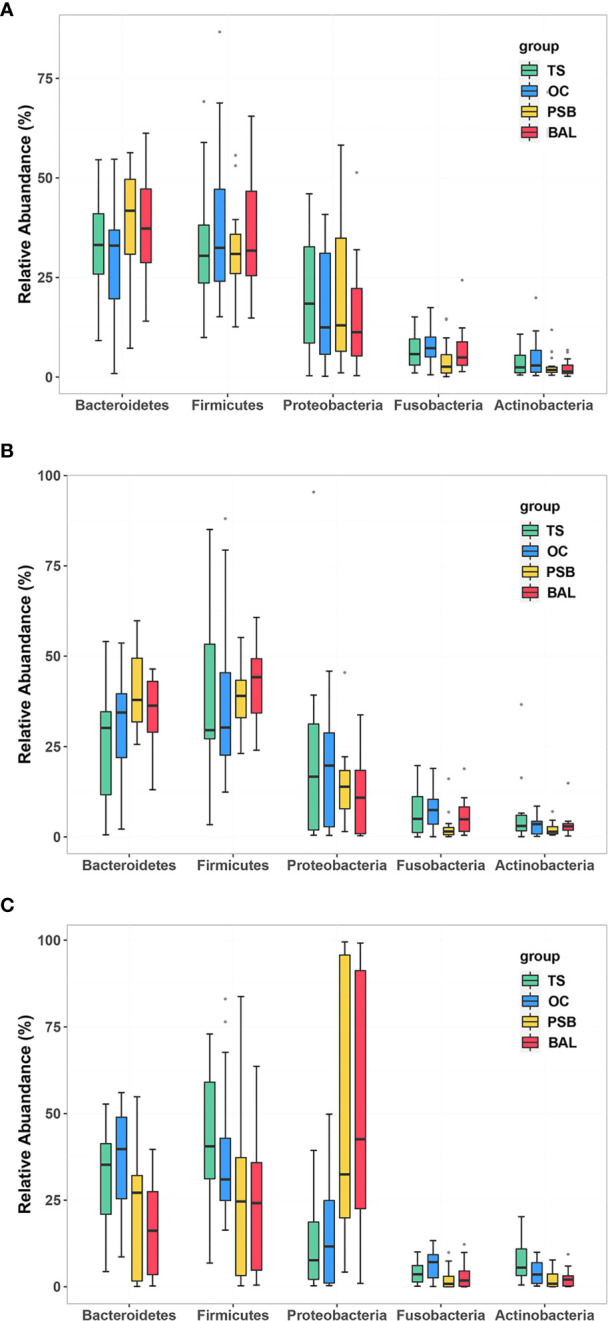
Relative abundance of phylum level OTUs at four distinct biogeographic sites for NMTT **(A)**, SGC **(B)**, and SCC **(C)** patients. Box plots show the relative abundance and significance of the most significant genera. The center point denotes the median. Points outside the whiskers represent outlier samples.

In all samples, the most well-represented phyla were Bacteroidetes, Firmicutes, Proteobacteria, and Fusobacteria, which accounted for ~90% of the total relative abundance (**Figure 1** and **Table S1**). The overall phylum level composition of microbiota significantly shifted at each of the four biogeographic sites in SCC patients, while no significant differences in composition were observed at any of the four sites in either NMTT or SGC patients (**Figure 1** and **Table S1**). More specifically, as the respiratory tract deepened (from throat to alveolus), the relative abundance of Bacteroidetes and Firmicutes gradually decreased, while the relative abundance of Proteobacteria gradually increased (**Table S1**).

### Comparison of Microbiota Composition and Effect Size Analysis

**Figure 2:**
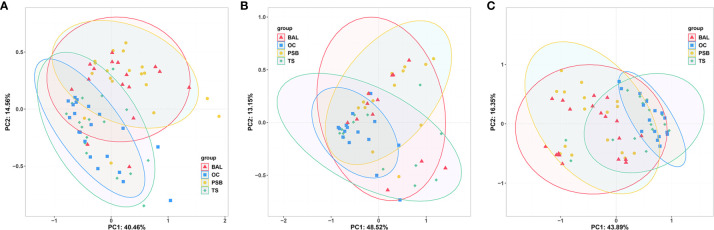
Unweighted UniFrac principal component analysis of microbiota differences among distinct biogeographic sites in NMTT **(A)**, SGC **(B)** and SCC **(C)** patients.

We then performed PCoA to investigate the potential differences in microbiota composition among different biogeographic sites for all three tracheal tumor types (**Figure 2**). In particular, OC microbiota appeared to explain 8.5%, 12.0%, and 11.4% of the variance in tracheal microbiota among NMTT, SGC, and SCC patients, respectively. Similarly, TS microbiota apparently explained 8.6%, 10.6%, and 14.1% of the variance in tracheal tumor microbiota for the NMTT, SGC, and SCC groups, respectively. In addition, the OC and TS microbiota explained 14.2% and 17.6% of the variance of microbiota in lung alveoli of SCC patients.

Genus level comparisons by Anosim and PERMANOVA showed no significant differences in microbiota composition at the OC and TS sites nor between the PSB and BALF samples in any of the patient groups, while the SGC group also had no significant differences between BALF communities and those in TS or OC samples. Further examination of PERMANOVA comparison of microbiota composition at the OC, TS, tracheal tumors, and lung alveoli sites revealed a significant correlation between the microbiota composition in PSB samples with those in TS and OC samples in all three groups (**Table 2**). These results indicated that the microbiota composition of OC and TS were significantly related with that of the LRT.

**Table 2:** Comparisons of microbiota at distinct biogeographic sites.

Disease	Site	PERMANOVA (R2)	*P* value	ANOSIM (R)	*P* value
NMTT	TS *vs* OC	0.014	0.806	-0.039	0.89
NMTT	TS *vs* PSB	0.085	0.002	0.090	0.005
NMTT	TS *vs* BAL	0.054	0.078	0.065	0.04
NMTT	OC *vs* PSB	0.086	0.001	0.136	0.002
NMTT	OC *vs* BAL	0.058	0.032	0.103	0.017
NMTT	PSB *vs* BAL	0.021	0.712	-0.035	0.888
SCC	TS *vs* OC	0.041	0.269	0.025	0.223
SCC	TS *vs* PSB	0.114	0.002	0.186	0.001
SCC	TS *vs* BAL	0.142	0.001	0.212	0.002
SCC	OC *vs* PSB	0.141	0.001	0.230	0.001
SCC	OC *vs* BAL	0.176	0.001	0.258	0.001
SCC	PSB *vs* BAL	0.012	0.939	-0.016	0.581
SGC	TS *vs* OC	0.025	0.644	-0.019	0.652
SGC	TS *vs* PSB	0.120	0.001	0.157	0.002
SGC	TS *vs* BAL	0.057	0.134	0.001	0.42
SGC	OC *vs* PSB	0.106	0.002	0.142	0.006
SGC	OC *vs* BAL	0.034	0.522	0.002	0.423
SGC	PSB *vs* BAL	0.078	0.077	0.044	0.139

Comparison of community similarity in different respiratory tract sites in patients with different tumor types using anosim function of vegan. Permutational multivariate analysis of variance (PERMANOVA) of community composition at different respiratory tract sites in three tumor type groups using adonis function of vegan.

### Microbial Diversity of Tracheal Tumors

In light of our above findings showing clear relationships between URT and LRT microbiota, we next examined microbial community richness using the ACE and Chao1 estimators and community diversity using the Shannon index. The results showed no significant differences among the four sites in any of the three pathological types (**Figure S1**). However, comparisons of α-diversity by Simpson index among all three groups revealed significant differences in their tracheal microbiota (*p*=0.018). Furthermore, SCC patients showed significantly lower α-diversity compared to SGC patients (Shannon index, *p*=0.028; Simpson index, *p*=0.009).

**Figure 3:**
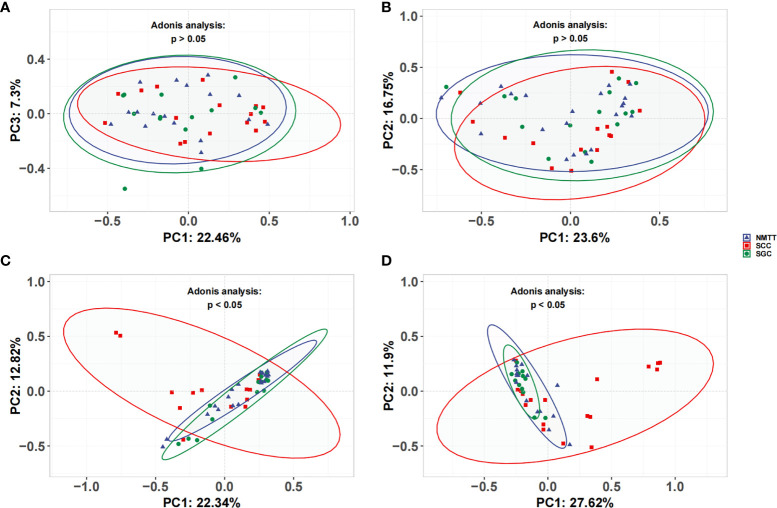
Unweighted UniFrac principal coordinate analysis of microbiota from **(A)** OC, **(B)** throat swab, **(C)** PSB, and **(D)** BALF samples between NMTT, SGC and SCC patients.

No significant differences were observed in the UniFrac distances between microbiota of OC and TS samples within each group (**Figures 3A, B**). β-diversity plots showed distinct separation of the tracheal microbiota of SCC subjects from those of SGC or NMTT (*p* < 0.05, **Figure 3C**). PC1 explained 22.34% of the variation among groups, while PC2 explained 12.82% of the variation. In BALF samples, PCoA plots showed that microbiota in SCC group samples were obviously distinct from those of the SGC group (*p* < 0.05, **Figure 3D**), with PC1 accounting for 11.9% of the variation, and PC2 accounting for 27.62% of the variation.

### Taxonomic Characteristics of Tracheal Tumor Microbiota at Different Sites

**Figure 4:**
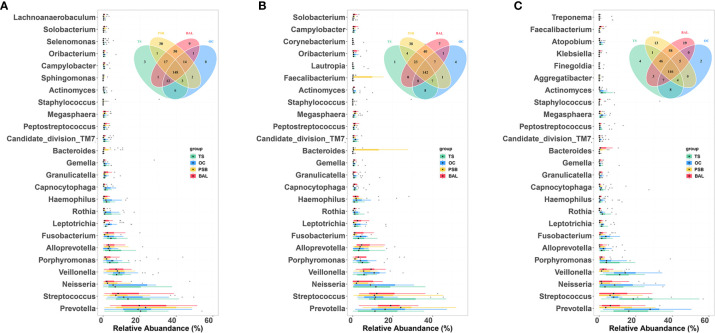
Comparison of predominant microbial genera at four biogeographic sites in the NMTT **(A)**, SGC **(B)** and SCC **(C)** groups. Box plots show the relative abundance and significance of the most significant genera in microbiota at each site. The center point denotes the median. Points outside the whiskers represent outlier samples. Venn diagram in each panel illustrates overlap of genera in microbiota among the OC, TS, trachea, and lung alveoli sites.

To determine which bacterial genera could serve as indicators for microbiota at different biogeographic sites, we investigated the relative abundance of genera which appeared at a frequency of 0.1% or greater. We found that most genera were shared by all four sites in different pathology groups, as shown by Venn diagram (**Figure 4** and **Table S2**). Across all samples, a total of 311 genera were detected, with the nineteen most abundant genera accounting for ~80% of the total OTUs including *Prevotella*, *Streptococcus*, *Neisseria*, *Veillonella*, *Porphyromonas*, *Alloprevotella*, *Fusobacterium*, *Leptotrichia*, *Rothia*, *Haemophilus*, *Capnocytophaga*, *Granulicatella*, *Gemella*, *Bacteroides*, *Candidate_division_TM7*, *Peptostreptococcus*, *Megasphaera*, *Staphylococcu*s, and *Actinomyces* (**Figure 4** and **Table S2**). **Figure 4** shows taxa that are unique or shared among NMTT, SGC, and SCC groups at each geographical site. Notably, four taxa were exclusively detected in the OC, one in the TS, 38 in the PSB, and seven in the BALF of SGC patients. By contrast, two taxa were exclusively found in the OC, four in the TS, 30 in the PSB, and 19 in the BALF of only SCC patients.

**Figure 5:**
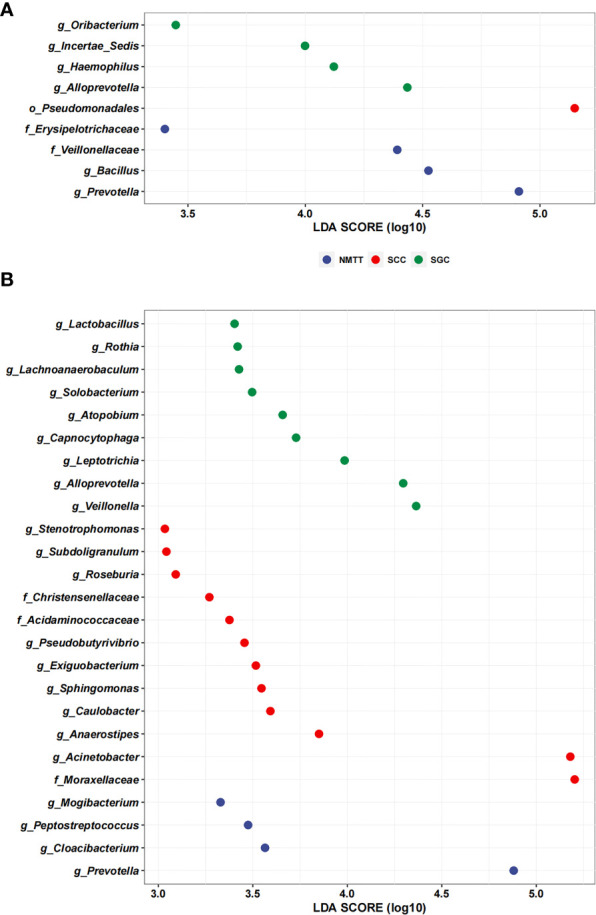
Linear discriminant analysis effect size (LefSe) based on operational taxonomic units (OTUs) characterize microbiomes of trachea **(A)**, and distal alveoli **(B)** between different pathological types of tracheal tumor groups.

We performed LEfSe analysis separately on the microbiota of different sampling sites to identify which taxa contribute the strongest effects to differences between the three groups. The results showed no significant differences between taxa in the OC and TS samples, whereas analysis of trachea microbiota revealed a relatively high abundance of genera *Oribacterium*, *Incertae Sedis*, *Haemophilus*, and *Alloprevotella* among samples from SGC patients (**Figure 5A**). In contrast, order Pseudomonadales and the Erysipelotrichaceae and Veillonellaceae families (which included *Bacillus* and *Prevotella*) were relatively enriched among SCC group samples (**Figure 5A**).

Further LEfSE analysis of distal alveoli microbiota showed significantly higher abundances of *Lactobacillus*, *Rothia*, *Lachnoanaerobaculum*, *Solobacterium*, *Atopobium*, *Capnocytophaga*, *Leptotrichia*, *Alloprevotella* and *Veillonella* in SCC patients, but higher abundances of *Stenotrophomonas*, *Subdoligranulum*, *Roseburia*, *Christensenellaceae*, *Acidaminococcaceae*, *Pseudobutyrivibrio*, *Exiguobacterium*, *Sphingomonas*, *Caulobacter*, *Anaerostipes*, *Acinetobacter*, and *Moraxellaceae* in SGC patients at the genus level (**Figure 5B**). Collectively, these results suggested that SCC and SGC appear to enrich for distinctly different sets of bacterial genera.

### Potential Bacterial Biomarkers for Tracheal Tumor Patients

**Figure 6:**
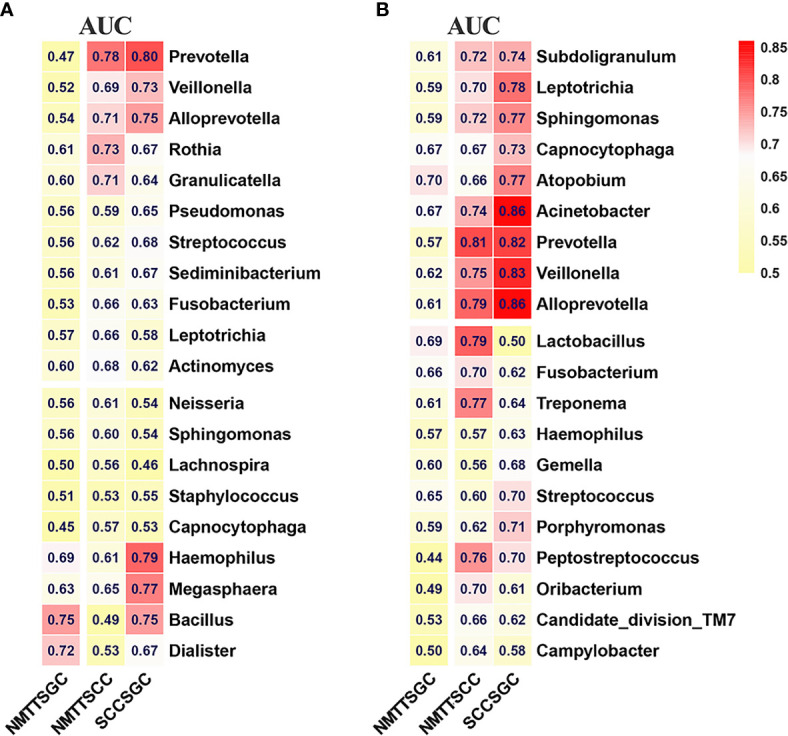
Differences in bacterial genera among NMTT, SGC, and SCC groups in trachea **(A)** and lung alveoli **(B)** sites. The receiver operating characteristic curves for classification were performed and the values of the area under the ROC curve (AUC) are shown in the heatmaps.

To identify candidate biomarkers for different tracheal tumor types, we performed random forest analysis to highlight differences in composition of microbiota between PSB and BALF samples. Biomarker screening was based on two criteria: candidates 1) were among the 50 features showing the greatest mean decrease in Gini importance by random forest analysis, and 2) had a relative abundance of greater than 0.1% among total bacterial genera. As shown in **Table S3**, we screened 20 potentially resolvable microbial genera from the PSB and BALF samples, respectively (n=40 total). The ROC analysis was further performed to validate the diagnostic ability of these potential biomarkers for classification of different tracheal tumors (**Figure 6**). In particular, *Prevotella* and *Alloprevotella* genera were found to serve as strong potential indicators among the classifiers. For example, at the tracheal site, *Prevotella* produced AUCs of 78% and 80% in distinguishing SCC from NMTT or SGC from patients, respectively, while *Alloprevotella* showed AUCs of 71% and 75% in distinguishing SCC from NMTT or SCC from SGC patients, respectively (**Figure 6A**). In addition, *Bacillus* had the potential ability to distinguish SGC patients from patients with NMTT (AUC = 75%) and SCC (AUC = 75%). Several genera in distal lung alveoli samples, especially *Prevotella*, *Alloprevotella*, *Acinetobater*, and *Veillonella*, also showed relatively strong ability to distinguish SCC from NMTT and SGC (**Figure 6B**), with AUCs ranging from 0.74-0.86. These results suggested that several genera were distinctly enriched at these biogeographic sites during SCC or SGC development, and could therefore be further developed as potential diagnostic markers for adoption in clinic.

## Discussion

To our knowledge, this report represents the first comprehensive investigation of the microbial composition and diversity of the microbiome at four different biogeographic sites (saliva, throat, trachea, and distal alveoli) in patients with PMTT. We found that the LRT microbiota are affected by oral and throat microbiota and observed that *Prevotella* and *Veillonella* species decrease in relative abundance with increasing depth into the airway. Our results also indicate that alpha diversity is lower in SCC patients compared to that in the SGC and NMTT groups. In addition, the relative abundance of *Prevotella* is lower in the SCC group than that in the SGC and NMTT groups, while *Alloprevotella* is found in the highest abundance in LRT samples of the SGC group. These significant differences in microbial diversity, and taxa abundance associated with different anatomical sites and pathological types suggests an imbalance in the microbiota of the PMTT microenvironment and implies that some taxa may participate in the pathology of PMTT.

Discrepancies in the results obtained by different studies could be attributable to differences in the number of cases, ethnicity, gender, smoking history, age, and inclusion criteria. We enrolled patients from our clinics in Shanghai and Beijing who originated from provinces throughout China to avoid geographical selection bias and employed strict inclusion and exclusion criteria to minimize the effects of confounding factors. Contaminant-controlled analysis framework in our study allowed the accurate detection of microbial DNA for sequence. We found that the airway and oral microbiota associated with tracheal tumors are primarily composed of four phyla, which is consistent with findings of previous studies (4, 27). Unlike NMTT and SGC patients, the abundance of Proteobacteria in the LRT is significantly elevated in SCC patients, while the other phyla have significantly lower relative abundance. This effect could be at least partially explained by impaired local immunity at the lesion site due to smoking, which causes massive proliferation of pathogenic bacteria. It should be noted that Proteobacteria, such as *Pseudomonas aeruginosa*, are reportedly involved in chronic lung disease, lung fibrosis, and asthma (28–30).

Studies in lung cancer patients and animal models have demonstrated tumor-associated dysregulation of airway microbiota, which can in turn impact cancer progression by shaping conditions in the tumor microenvironment and modulating the activity of tumor-infiltrating immune cells (10–15, 31). Our previous study found that lung cancer-associated microbiota profile is distinct from that found in healthy controls, and the altered cancer-associated microbiota is not restricted to tumor tissue (10). These results suggested considerable spatial heterogeneity in bacterial community composition within the lungs of lung cancer patients. However, the role of airway microbiota in PMTT remains largely unknown. The respiratory tract is a complex system that is exposed to a large number of airborne microorganisms and is inhabited by niche-specific communities of microbes. Moreover, the oral microbiota shape the respiratory microbiome (32), which may contribute to the carcinogenesis of PMTT *via* mucosal dispersion and microaspiration of oral bacteria and their metabolites to the respiratory tract. Dysbiosis of oral microbiota is closely associated with cancer, including lung, esophageal, and colorectal cancer (33–37). For instance, the abundance of the periodontal pathogen *Porphyromonas gingivalis* is accompanied by an elevated risk of esophageal squamous cell carcinoma (33). Other work has suggested that *Peptococcus* and *Centipeda* species in saliva and mucosal *Subdoligranulum* species could serve as drivers of tumorigenesis of colorectal cancer (34). Dysbiosis of the salivary microbiome is also associated with non-smoking lung cancer and correlated with systemic inflammatory marker (35).

In this study, we found that the microbiota in the OC and TS could explain 8.5%-17.6% of the variance of the lower respiratory tract microbiota. Interestingly, we found that the microbiota of OC and throat exert a statistically significant effect on microbiota of the trachea but not on distal alveoli in SGC patients. However, in SCC patients, the microbiota of the OC and throat significantly affect the tracheal and distal alveolar microbiota, and similar results were also observed in NMTT patients. SGC are most commonly found in oral cancer patients, but is rare in lung cancer patients. Although rare, tracheal SGC also originates from tumorigenesis of the minor salivary glands located in the mucous membranes of the head and neck. Tracheal SGC microbiota can be thus affected by the oral microbiota originating in the adjacent oral cavity or from submucosal salivary glands that extend down into the trachea.

In healthy individuals, the lung microbiome is generally believed to be inoculated by OC bacteria, and that the community is maintained by a balance between immigration, colonization, and competition processes (32). However, this balance is apparently disturbed during several lung diseases (5). This likelihood is supported by our finding of decreased alpha diversity in SCC patients compared with that in SGC and NMTT subjects. However, we found no consistent changes in microbial diversity of distal lung alveoli and oral cavity among different carcinoma subtypes. Reduced microbial diversity has been considered a feature of some cancers, such as esophageal adenocarcinoma, gastric carcinoma, and colorectal cancer. Our previous study showed that decreased microbial diversity in the distal lung alveoli is associated with increased risk of lung cancer (10).

The genera *Prevotella* and *Veillonella* are among the most abundant bacteria in the respiratory tract of healthy individuals and are universal commensal colonizers of mucosal surfaces in a “healthy” lung microbiome (38–40). Recent work has shown that the relative abundance of *Prevotella* in BALF is increased in lung cancer patients (11). Here, we also observed that the relative abundance of *Prevotella* increased in the SCC group. *Prevotella* can adhere to epithelial cells and form a biofilm through their ability to modulate host immune response and metabolic activity (41). Alternatively, Segal et al. classified the microbiota of BALF samples from healthy subjects into two types: a supraglottic predominant taxa (SPT) pneumotype and a background predominant taxa (BPT) pneumotype (42, 43). Further studies revealed that characteristic SPT taxa, including *Prevotella* and *Veillonella*, were positively correlated with multiple cytokine levels, including Th17 cytokines such as IL-1α, IL-1β, IL-6, fractalkine, and IL-17, in both Th17 cells and neutrophils (43). Thus, URT-derived bacteria and/or their metabolites in the lung can regulate the tonic level of airway inflammation and Th17 immune activation (43). Tsay et al. found that lower airway dysbiosis induced by microaspiration of oral commensals affect lung tumorigenesis by promoting an IL-17 driven inflammatory phenotype (12). Dysregulation of local lung microbiota stimulated Myd88-dependent IL-1β and IL-23 production from myeloid cells, inducing proliferation and activation of Vγ6+Vδ1+ γδ T cells that produced IL-17 and other effector molecules to promote inflammation and tumorigenesis (31).

Among these URT bacteria, *Veillonella* is associated with increased mucosal inflammation in the airway, and is significantly more abundant in saliva and BALF samples from lung cancer patients than healthy control subjects (14, 15). Airway microbiota can regulate specific oncogenic pathways that directly drive carcinogenesis. *Veillonella* is also associated with transcriptomic changes in airway epithelial cells, especially with activation of the ERK/PI3K pathway relevant to lung cancer (13). In addition to *Veillonella*, our results reveal that *Alloprevotella* is found in the highest abundance in LRT samples of the SGC group, suggesting a strong association between SGC and predominant oral bacteria. Other studies have shown that *Alloprevotella* likely contributes to the enrichment for proinflammatory genes, has been reported to be progressively enriched along the order of negative control → precancerous lesions → oral cancer (37). Our results are thus consistent with previous studies and further indicate that specific bacterial taxa may serve as potential biomarkers and new therapeutic targets.

Some limitations of this study should be noted. First, for ethical reasons, we did not obtain PSB or BALF samples from healthy individuals as controls, so the use of specific bacteria to distinguish different tracheal tumors types may be unreliable in some cases, resulting in false positive correlations. Second, respiratory microbial communities undergo dynamic changes, and cross-sectional studies may not reflect a comprehensive landscape of microbial features across all stages of the disease. Third, this pilot study utilizes a small cohort and therefore requires a large-scale longitudinal cohort for validation. In addition, the sample size is too small to perform risk stratification or to predict prognosis.

## Conclusion

In this study, we investigated the composition, diversity, and differences in respiratory tract microbiota at different sites in PMTT patients. This study uncovers novel and plausible correlations between dysbiosis in microbiota and PMTT. This work also identified bacterial genera that are unique to specific sites during SCC or SGC development and can therefore serve as candidate targets for PMTT diagnosis or intervention, although large sample studies are needed to validate their reliability as biomarkers.

## Data Availability Statement

The datasets presented in this study can be found in online repositories. The names of the repository/repositories and accession number(s) can be found below: https://www.ncbi.nlm.nih.gov/genbank/, PRJNA766788.

## Ethics Statement

The studies involving human participants were reviewed and approved by the Ethics Committee of Ruijin Hospital Affiliated with Shanghai Jiaotong University and Emergency General Hospital. The patients/participants provided their written informed consent to participate in this study. Written informed consent was obtained from the individual(s) for the publication of any potentially identifiable images or data included in this article.

## Author Contributions

K-XL and H-XL: collection and assembly of data, data analysis and interpretation, writing the original draft, review, and editing. JZ, NZ, Y-ZZ, and M-MT: Sample and data collection, data analysis. J-MQ and H-WW conception and design and administrative support, review, and editing. All authors contributed to the article and approved the submitted version.

## Funding

This work was supported by grants from Shanghai Key Laboratory of Emergency Prevention, Diagnosis and Treatment of Respiratory Infectious Disease (20dz2261100), the National Natural Science Foundation of China (81570066 and 81630001), Shanghai Top-Priority Clinical Key Disciplines Construction Project (2017ZZ02014), Shanghai Shenkang Hospital Development Center Clinical Science and Technology Innovation Project (SHDC12018102), National Key Research & Development Program of China (2018YFE0102400), National Innovative Research Team of High-level Local Universities in Shanghai, Shanghai Municipal Key Clinical Specialty (shslczdzk02202) and the Young and Middle-aged Backbone Talent training plan of Fujian Provincial Health Commission (2020GGA053).

## Conflict of Interest

The authors declare that the research was conducted in the absence of any commercial or financial relationships that could be construed as a potential conflict of interest.

## Publisher’s Note

All claims expressed in this article are solely those of the authors and do not necessarily represent those of their affiliated organizations, or those of the publisher, the editors and the reviewers. Any product that may be evaluated in this article, or claim that may be made by its manufacturer, is not guaranteed or endorsed by the publisher.

## Supplementary Material

Supplementary Figure 1 Alpha-Diversity at the genus level estimated by ACE, Chao1, and Shannon estimator for NMTT **(A)**, SGC **(B)**, and SCC **(C)** groups.
