# Supplementary figures and images for: Biogeography of the Respiratory Tract Microbiome in Patients With Malignant Tracheal Tumors
Source: Front Oncol. 2021 Nov 19;11:758917. doi: 10.3389/fonc.2021.758917 (PMC8640173; doi:10.3389/fonc.2021.758917)

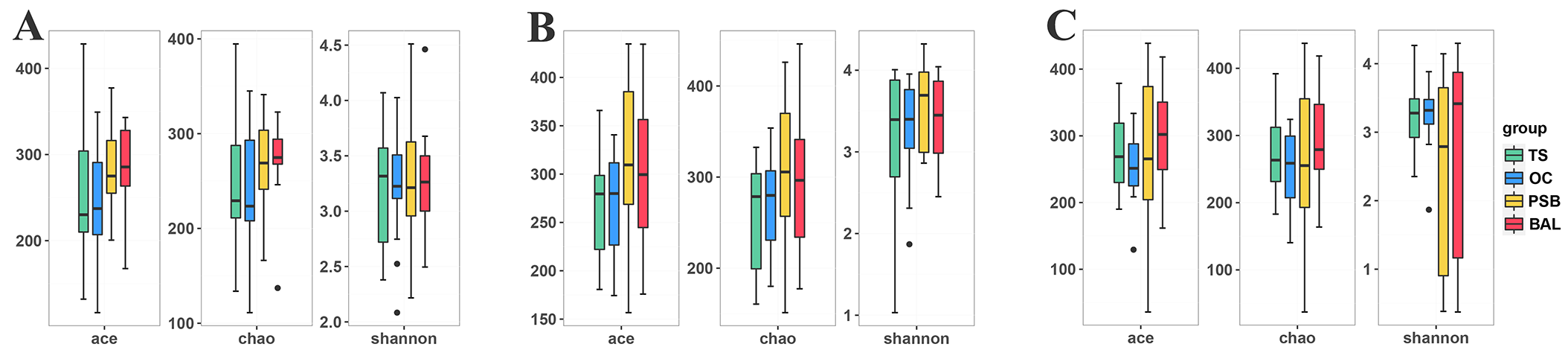

Supplement: Supplementary Figure 1 — Alpha-Diversity at the genus level estimated by ACE, Chao1, and Shannon estimator for NMTT (A), SGC (B), and SCC (C) groups. [file Image_1.tif]
